# Health literacy, health status, and healthcare utilization of taiwanese adults: results from a national survey

**DOI:** 10.1186/1471-2458-10-614

**Published:** 2010-10-16

**Authors:** Shoou-Yih D Lee, Tzu-I Tsai, Yi-Wen Tsai, Ken N Kuo

**Affiliations:** 1 Department of Health Management and Policy, School of Public Health, University of Michigan, 1420 Washington Heights, Ann Arbor, MI 48109-2029, USA; 2 School of Nursing, National Yang-Ming University, No.155, Sec.2, Linong Street, Taipei, 112, Taiwan; 3 Institute of Health and Welfare Policy, National Yang-Ming University, No.155, Sec.2, Linong Street, Taipei, 112, Taiwan; 4 Health Policy Research and Development, Institute of Population Health Sciences, National Health Research Institutes, 35, Keyan Road, Zhunan Town, Miaoli County, 350, Taiwan

## Abstract

**Background:**

Low health literacy is considered a worldwide health threat. The purpose of this study is to assess the prevalence and socio-demographic covariates of low health literacy in Taiwanese adults and to investigate the relationships between health literacy and health status and health care utilization.

**Methods:**

A national survey of 1493 adults was conducted in 2008. Health literacy was measured using the Mandarin Health Literacy Scale. Health status was measured based on self-rated physical and mental health. Health care utilization was measured based on self-reported outpatient clinic visits, emergency room visits, and hospitalizations.

**Results:**

Approximately thirty percent of adults were found to have low (inadequate or marginal) health literacy. They tended to be older, have fewer years of schooling, lower household income, and reside in less populated areas. Inadequate health literacy was associated with poorer mental health (OR, 0.57; 95% CI, 0.35-0.91). No association was found between health literacy and health care utilization even after adjusting for other covariates.

**Conclusions:**

Low (inadequate and marginal) health literacy is prevalent in Taiwan. High prevalence of low health literacy is not necessarily indicative of the need for interventions. Systematic efforts to evaluate the impact of low health literacy on health outcomes in other countries would help to illuminate features of health care delivery and financing systems that may mitigate the adverse health effects of low health literacy.

## Background

Low health literacy, defined as an individual's limited ability to obtain, process, and understand basic health information and services needed to make appropriate health decisions [1], has been suggested as a worldwide problem and a global challenge for the 21st century [2]. In its recent report, the WHO Commission on the Social Determinants of Health (CSDH) declares health literacy as a major determinant for health and advises countries to create a multi-stakeholder Council on Health Literacy to monitor and coordinate strategic activities to enhance health literacy [3].

The declaration of health literacy as a global challenge and the CSDH's recommendation of health literacy initiatives as important elements in the strategies to reduce health inequity are largely based on studies in the United States and a few other English-speaking countries. Those studies indicated a high prevalence of low health literacy in adults and found that low health literacy was linked to limited understanding of health information and medical instructions [4-7], inadequate self-management of diseases [6], underuse of preventive services and routine physician visits [8-13], increased hospitalizations and medical costs [14,15], and high mortality rates [16]. So far, little research on health literacy has been conducted in non-English-speaking countries. It is unclear if low health literacy is a worldwide problem and whether low health literacy also has adverse health effects in countries that have more equitable access to health care.

In this paper, we report the results of a national assessment of health literacy in Taiwanese adults. The purpose is twofold: (1) to assess the prevalence and distribution of low health literacy in Taiwanese adults, and (2) to examine the associations of low health literacy with self-rated physical and mental health status and health care utilization (outpatient clinic visits, hospitalization, and emergency room visits). The results would help to confirm if low health literacy is indeed a worldwide health threat, even in a country where the literacy rate is 97.8% and where 22.48% of the adult population has a university degree [17]. Furthermore, we could learn how health literacy contributes to health status and health care utilization in an equitable health care system. Implemented in 1995, Taiwan's national health insurance system has increased the coverage from 57% to 98% of the population. The system has also expanded access by waiving copayments for the very poor, veterans, and aboriginal populations [18].

## Methods

### Sample

In 2008, a survey was conducted to assess health literacy in a national sample of Taiwanese adults using the Mandarin Health Literacy Scale (MHLS) [19]. Subjects in the survey were selected based on the Taiwanese household registration system and using a multi-stage stratified, probability-proportional-to-size sampling strategy [20]. A total of 1,967 adults aged 18 and older were selected and contacted by mail or phone for participation in the survey. Excluding non-responders (subjects who refused or could not be reached after five attempts of contact) and subjects who had uncorrectable vision and hearing problems and who were cognitively impaired, 1,492 adults voluntarily participated in the survey. The response rate was 75.8%. Respondents and non-respondents were not different statistically in terms of age and gender, and formal education.

### Ethical Considerations

The protocol for the national survey was approved by the institutional review board at the National Health Research Institutes in Taiwan. In the letter and the phone message to potential participants, we explained the purpose of the survey and asked for their voluntary participation. Those who agreed to participate were scheduled for an in-person interview conducted by a trained interviewer. Before the interview, the interviewer first explained the purpose of the survey, the study participants' rights, the risk and benefit of participation, and our plan to protect the confidentiality of study participants. Further, a signed informed consent was obtained prior to the interview.

### Data Collection

Previous research suggests that illiterate subjects may feel embarrassed about not being able to read and may be uncomfortable taking the self-administered health literacy test, which requires the respondent to read and answer a battery of health related questions [21,22]. To avoid embarrassment, at the beginning of the interview, we asked the respondents to read aloud a brief text as a way to identify those who were illiterate or unable to read. Those respondents (N = 162) who were unable to read were not asked to complete the self-administered health literacy test and received a zero score. The remaining respondents took the health literacy test and were scored according to their performance on the test. All respondents, whether or not they took the health literacy test, answered all the remaining survey questions, administered by an interviewer, regarding socio-demographic attributes, health status, and healthcare utilization. On average, the interview took around 40 minutes to complete.

### Measurement

The MHLS is a reading and numeracy instrument designed to assess health literacy in Mandarin Chinese or Standard Chinese [19]. The scale contains 50 items, of which 33 test the comprehension of health-related texts and 17 assess numeracy skills. In a random sample of 323 Taiwanese adults, the scale was found to have a high correlation with years of formal education, suggesting high convergent validity. It was significantly associated with reading habit, health knowledge, and receipt of assistance with reading written health materials, indicating good predictive validity. Furthermore, it had high internal reliability (Cronbach's alpha = 0.95) and split-half reliability (Spearman-Brown correction = 0.95). In the current study sample, the internal reliability of the scale was 0.88. Following Tsai and colleagues [19], we classified the respondents into three health literacy levels: inadequate (0-30), marginal (31-42), and adequate (43-50).

Socio-demographic attributes included age, gender, educational attainment (years of formal schooling), household income, and residential location (metropolitan city, mid-size city, small city, rural/remote area). Health status was assessed by asking respondents to self-rate their physical and mental health over the past six months on a 5-point Likert scale, ranging from 1 (very poor) to 5 (excellent). Health care utilization was measured by asking respondents to answer yes or no to (1) whether they had at least an outpatient visit in the previous 3 months, (2) whether they ever visited an emergency room (ER) in the last year, and (3) whether they were ever hospitalized in the last year.

### Statistical Analysis

Descriptive statistics (mean, SD, and percentage) were performed to examine the level of health literacy in the sample as a whole and by socio-demographic attributes. For descriptive analysis, age was classified into 18-24 years, 25-39 years, 40-49 years, 50-64 years, and ≥65 years; educational attainment: 0 year of formal schooling, 1-6 years, 7-12 years, 13-16 years, and ≥17 years; prior year's average household income: ≤50% national average (≤NT$461,937 or ≤US$14,435.5), 51%-75% national average (NT$461,938-NT$692,906 or US$14,435.6-US$21,653.3), 76%-100% national average (NT$692,907-NT$923,875 or US$21,653.4-US$28,871.1), >100% national average (>NT$923,875 or >US$28,871.1). Significant differences in health literacy across socio-demographic groups were evaluated using the chi-square (χ^2^) test. The associations of health literacy with health status and healthcare utilization were assessed using the χ^2 ^test and multinomial logit modeling. The logit models controlled for age, gender, educational attainment, household income, and residential location. For the logit models on health care utilization, physical health status and mental health status were also included as controls. The interval form of age and educational attainment was used in the multinomial logit models to conserve statistical power. All statistical analysis was performed using the Statistical Analysis Software package, SAS version 9.1.

## Results

### Sociodemographic Characteristics of the sample

Respondents ranged in age from 18.6 to 92.8 years, with a mean age of 46.3. The gender distribution was about equal. The largest group of respondents had 7-12 years of formal schooling (40.0%) and the second largest had 13-16 years (32.2%). Three hundred and thirteen respondents (21.0%) did not report household income; 22.9% of respondents were in households that earned less than 50% of the national average household income in 2007, 16.9% earned 51-75% of the average, 12.5% earned 76-100% of the average, and 26.3% earned more than 100% of the average. The majority of respondents (58.7%) resided in metropolitan and mid-size cities, 21.7% in small cities, and 19.6% in rural/remote areas (Table 1).

**Table 1:** Socio-demographic Attributes and Health Literacy Level of a National Sample of Adults in Taiwan, 2008

	N	%	MHLS Score	Inadequate HL		Marginal HL		Adequate HL		**X**^**2**^**-test** p-value
			mean (SD)	N	%	N	%	N	%	
**Entire Sample**	1493	100.0	39.2 (14,8)	205	13.7	247	16.5	1041	69.7	
**Age**										<0.001
18-24 years	149	10.0	45.0 (6.2)	4	2.7	16	10.7	129	86.6	
25-39 years	456	30.5	45.0 (6.6)	12	2.6	48	10.5	396	86.8	
40-49 years	292	19.6	43.9 (7.1)	10	3.4	47	16.1	235	80.5	
50-64 years	358	24.0	38.1 (14.8)	52	14.5	80	22.4	226	63.1	
≥65 years	237	15.9	20.6 (20.2)	126	53.2	56	23.6	53	23.2	
**Gender**										0.07
Male	736	49.3	40.0 (13.5)	91	12.4	136	18.5	509	69.2	
Female	757	50.7	38.5 (15.9)	114	15.1	111	14.7	532	70.3	
**Years of Schooling**										<0.001
0 year	105	7.0	0.3 (3.4)	104	99.0	1	0.9	0	0.0	
1-6 years	246	16.5	30.3 (17.6)	73	29.7	90	36.6	83	33.7	
7-12 years	597	40.0	42.8 (6.8)	28	4.7	131	21.9	438	73.4	
13-16 years	481	32.2	46.7 (2.8)	0	0.0	24	5.0	457	95.0	
≥17 years	64	4.3	48.0 (2.4)	0	0.0	1	1.6	63	98.4	
**Household Income**^**a**^										<0.001^b^
≤50% of average	342	22.9	32.4 (18.4)	93	27.2	81	23.7	168	49.1	
51-75% of average	252	16.9	41.4 (11.6)	23	9.1	44	17.5	185	73.4	
76-100% of average	193	12.9	43.6 (9.4)	10	5.2	23	11.9	160	82.9	
>100% of average	393	26.3	45.3 (6.5)	7	1.8	40	10.2	346	88.0	
Missing	313	21.0	34.7(18.0)	72	23.0	59	18.9	182	58.2	
**Residential Location**										<0.001
Metropolitan city	367	24.6	42.0 (12.4)	28	7.6	50	13.6	289	78.8	
Mid-size city	509	34.1	41.2 (12.1)	45	8.8	96	18.9	368	72.3	
Small city	324	21.7	38.8 (15.0)	48	14.8	52	16.1	224	69.1	
Rural/remote area	292	19.6	32.7 (19.1)	84	28.8	49	16.8	159	54.4	

^a ^The national average of annual household income in 2007 was around NT$923,875. (Source: Directorate General of Budget, Accounting and Statistics, Executive Yuan. Report on the Survey of Family Income and Expenditure in Taiwan Area of Republic of China.).

### Health literacy and socio-demographic factors

The mean MHLS score of the sample was 39.2. The health literacy level of 69.7% respondents was considered to be adequate, 16.6% marginal, and 13.7% inadequate. The χ^2 ^analysis indicated significant variation in health literacy by age, educational attainment, household income, and residential location. In general, the level of health literacy was lower among adults with older age, fewer years of formal schooling, lower household income, and living in less populated areas. Although males appeared to have a higher average MHLS score than females, the difference was not statistically significant (Table 1).

### Health literacy, health status and health service utilization

The majority of respondents perceived their physical and mental health to be average or better. Around 64% of respondents reported at least one outpatient visit in the past 3 months, 13.3% had visited an ER, and 6.9% had been hospitalized in the previous year (Table 2). The χ^2 ^tests showed that health literacy level was positively associated with self-rated physical and mental health status and negatively associated with outpatient visits and hospitalization. No significant association was found between health literacy level and ER utilization (Table 2).

**Table 2:** Health Status, Health Care Utilization, and Health Literacy in a National Sample of Adults in Taiwan, 2008

	N	%	Inadequate HL		Marginal HL		Adequate HL		**X**^**2**^**-test** p-value
			N	%	N	%	N	%	
**Self-Reported Physical Health**									<0.001^a^
Very poor	25	1.7	7	3.4	6	2.4	12	1.2	
Poor	175	11.7	42	20.5	28	11.3	105	11.1	
Average	756	50.6	99	48.3	125	50.6	532	51.1	
Good	343	23.0	39	19.0	54	21.9	250	24.0	
Excellent	194	13.0	18	8.8	34	13.8	142	13.6	
**Self-Reported Mental Health**									<0.01^a^
Very poor	15	1.0	4	2.0	3	1.2	8	0.8	
Poor	176	11.8	40	19.5	26	10.5	110	10.6	
Average	593	39.7	78	38.0	100	40.5	415	39.9	
Good	452	30.3	63	30.7	75	30.4	314	30.2	
Excellent	257	17.2	20	9.8	43	17.4	194	18.6	
**Outpatient Clinic Visit**									<0.01
Yes	957	64.1	152	74.2	157	63.6	648	62.2	
No	536	35.9	53	25.8	90	36.4	393	37.8	
**Emergency Room Visit**									0.277
Yes	198	13.3	34	16.6	34	13.8	130	12.5	
No	1295	86.7	171	83.4	213	86.2	911	87.5	
**Hospitalization**									<0.01
Yes	103	6.9	25	12.2	13	5.3	65	6.2	
No	1390	93.1	180	87.8	234	94.7	976	93.8	

^a ^Yates' correction was used to adjust for small observations in some of the cells.

Excluding 313 observations with missing information on household income, multinomial logit models were performed on 1,180 observations to further examine the associations of health literacy level with self-rated health status (physical and mental health) and health care utilization (outpatient clinic visit, ER visit, and hospitalization). Two dummy variables (marginal health literacy and inadequate health literacy) were included in the models to represent health literacy level. As results in Table 3 show, health literacy level, in general, was not significantly associated with self-rated health status or health care utilization. There was one exception: compared to those with adequate health literacy, Taiwanese adults with inadequate health literacy had poorer self-rated mental health (odds ratio = 0.62; 95% confidence interval = 0.39-0.99).

**Table 3:** Multivariate Estimation of the Association of Health Literacy with Health Status and Health Care Utilization (N = 1180)

	Physical Health		Mental Health		Outpatient clinic visit		ER visit		Hospitalization	
	OR	(95% CI)	OR	(95% CI)	OR	(95% CI)	OR	(95% CI)	OR	(95% CI)
Health Literacy^a^										
Marginal	1.02	(0.74-1.42)	1.05	(0.77-1.44)	0.89	(0.61-1.29)	1.15	(0.70-1.91)	0.60	(0.27-1.32)
Inadequate	0.82	(0.50-1.33)	0.62*	(0.39-0.99)	0.79	(0.45-1.39)	1.08	(0.51-2.27)	1.22	(0.47-3.06)
Age (years)^b^	1.00	(0.99-1.01)	1.01*	(1.00-1.02)	1.03*	(1.02-1.04)	1.01	(0.99-1.02)	1.13*	(1.01-1.06)
Male^c^	1.32*	(1.06-1.64)	1.08	(0.87-1.33)	0.68*	(0.52-0.87)	0.97	(0.68-1.38)	0.94	(0.57-1.55)
Education (years)^b^	1.03	(0.99-1.07)	1.02	(0.98-1.06)	1.03	(0.98-1.08)	1.02	(0.96-1.09)	1.09*	(1.00-1.18)
Household Income^d^										
51-75% of average	1.25	(0.91-1.73)	1.21	(0.88-1.65)	1.12	(0.77-1.62)	0.96	(0.58-1.58)	0.78	(0.39-1.56)
76-100% of average	1.33	(0.93-1.89)	1.50*	(1.06-2.11)	1.15	(0.76-1.74)	0.85	(0.48-1.50)	0.90	(0.43-1.91)
>100% of average	1.52*	(1.10-2.09)	1.36*	(1.00-1.85)	1.22	(0.84-1.76)	0.0	(0.48-1.34)	0.44*	(0.20-0.93)
Residential Location^e^										
Mid-size city	0.98	(0.73-1.32)	0.91	(0.68-1.22)	0.94	(0.67-1.31)	0.88	(0.54-1.43)	1.18	(0.57-2.46)
Small-size city	1.00	(0.72-1.40)	0.88	(0.64-1.23)	1.32	(0.89-1.96)	1.01	(0.59-1.73)	1.21	(0.54-2.70)
Rural/remote area	0.89	(0.63-1.27)	0.77	(0.54-1.08)	1.60*	(1.06-2.42)	1.05	(0.60-1.82)	1.98	(0.91-4.33)
Physical Health^f^										
Poor					0.53	(0.10-2.75)	0.38	(0.12-1.21)	0.15*	(0.04-0.58)
Average					0.24	(0.05-1.21)	0.26*	(0.08-0.83)	0.06*	(0.01-0.22)
Good					0.14*	(0.03-0.74)	0.13*	(0.04-0.45)	0.06*	(0.01-0.26)
Excellent					0.09*	(0.02-0.51)	0.16*	(0.04-0.62)	0.04*	(0.01-0.20)
Mental Health^f^										
Poor					0.55	(0.10-3.16)	1.23	(0.28-5.46)	4.49	(0.59-34.11)
Average					0.70	(0.12-4.02)	0.90	(0.20-4.12)	4.42	(0.55-35.56)
Good					0.82	(0.14-4.79)	1.21	(0.26-5.65)	2.84	(0.33-24.40)
Excellent					0.79	(0.13-4.70)	0.94	(0.18-4.80)	4.97	(0.53-47.01)

* p <0.05.

^a ^"Adequate health literacy" is the comparison.

^b ^The interval form of the variable, measured in year, is used.

^c ^"Female" is the comparison.

^d ^"≤50% of average" is the comparison.

^e ^"Metropolitcan city" is the comparison.

^f ^"Very poor" is the comparison.

Several covariates were significantly associated with self-rated health status and health care utilization. Older adults were more likely to report better mental health and were less likely to visit an outpatient clinic. Males, compared to females, were more likely to report better physical health and less use of outpatient clinics. Adults with higher educational attainment were more likely to be hospitalized in the previous year. Compared to adults in the lowest household income category, those in the highest household income level were more likely to report better physical and mental health and were less likely to be hospitalized in the last year. Adults with better physical health were less likely to use any of the health services examined in the study.

To further explore the findings, step-wise logit models were conducted (results are available from the corresponding author upon request). The purpose of the analysis was to determine which of the control variables explained the bivariate correlations between health literacy and health status and health care utilization. To perform the step-wise logit models, we first entered the two dummy variables of health literacy, and then added one at a time, iteratively, each of the control variables, to see at which point the significant correlation between health literacy and health status and health care utilization became non-significant. Results indicated that educational attainment accounted for the association between health literacy and physical health status. Three covariates--age, educational attainment, and household income--together explained the correlations between health literacy and health care utilization.

The multinomial logit results may be biased because of the deletion of 313 observations without household income information. To examine the potential bias, a separate set of multinomial logit models was conducted with imputed household income using linear regression estimation (covariates included age, gender, educational attainment, and residential location). The results were similar, suggesting that the deletion of those 313 observations did not affect the substantive interpretation of findings (results are available upon request).

## Discussion

In a population-based, nationally representative sample of Taiwanese adults, approximately 30% had either inadequate or marginal health literacy. By comparison, 36% of American adults surveyed in the 2003 National Assessment of Adult Literacy (NAAL) had below basic (can perform simple everyday literacy activities) and basic health literacy (can perform no more than the most simple and concrete literacy activities) [23]; and 11.4% and 15.5% of adults in the U.K. and Japan, respectively, were considered to have low health literacy in national surveys [13,24]. However, this comparison is imprecise, because no similar tests have been developed to allow for international comparison. For example, instead of using a scale, the Japanese survey measured health literacy based on a single-item screening question, "How confident are you filling out forms by yourself?," possibly under-estimating the percentage of adults with low health literacy.

Similar to findings in the NAAL [21] and previous health literacy studies [4,13,23,25-28], older age, poorer educational attainment, and lower income were found to be associated with lower levels of health literacy in Taiwan. Furthermore, Taiwanese adults living in less populated and more rural areas tended to have a lower level of health literacy than adults living in more populated and more urban areas. Taken together, these findings suggest low health literacy is not only a personal limitation but also an indication of disadvantaged social status.

Contrary to our expectation, health literacy was not associated with self-rated physical health after controlling for age, gender, educational attainment, household income, and residential location. Although inadequate health literacy was significantly correlated with poor self-rated mental health, the association between health literacy and self-rated mental health was limited in the multinomial logit model. These findings are surprising because most studies have shown a significant relationship between health literacy and self-rated health status [25,26,29-31]. One possible explanation for the difference is that previous studies were conducted primarily on patients recruited from clinics or older adults enrolled in a managed care plan; these patients may in general have poorer health than the general adult population. Alternatively, the limited contribution of health literacy to health status, over and beyond that of educational attainment, may be due to a high correspondence between health literacy and educational attainment in Taiwanese adults. If this is true, educational attainment would be a sufficient indicator of health literacy skills among Taiwanese adults and health literacy assessment would have limited value. Furthermore, a focus on reducing educational disparities in health may help to decrease the health burden of poor health literacy as well.

Also interesting was our findings that health literacy was not independently associated with health care utilization among Taiwanese adults. Current evidence on health literacy as a predictor for health care utilization is decidedly mixed. Baker and colleagues found that patients in outpatient clinics who had inadequate health literacy were more likely to have physician visits and be hospitalized [14,29], while other studies did not find health literacy to be associated with physician visits, use of ER, or hospitalization [4,32]. Based on our step-wise analysis results that age, educational attainment and household income jointly explained the associations between health literacy and health care utilization, we suspect that the inconsistencies observed in existing literature may result from inadequate consideration of socio-demographic factors. Alternatively, the finding of no association between health literacy and health care utilization may be due to the fact that needed health care is affordable and accessible to around 98% of the population in Taiwan.

Several study limitations are worth noting. The limitations also point to opportunities for future research. First, only self-rated measures of health status and health care utilization were examined in the study. The reliability of self-rated health care utilization, in particular, may be subject to recall bias or memory failure. Linking health literacy test results to medical claims data would provide a more reliable assessment of the consequences of low health literacy. Second, results reported in the study were obtained from a cross-sectional survey and no causality is established between health literacy and health status and health care utilization. A longitudinal study that follows the study sample and reassesses their health outcomes at a later time would help to discern the causal effects of health literacy. Third, the MHLS, similar to other health literacy assessments, is primarily a reading and comprehension test. It offers no indication about the respondent's communication skills, which may be equally important in determining an individual's ability to effectively navigate today's complex health care system. Fourth, a significant proportion of the sample was missing household income information. This may introduce systematic bias in the multinomial logit models. Although a separate analysis was conducted to assess the bias using imputed household income, the results should be interpreted with caution. Finally, we did not examine the associations of health literacy with disease knowledge, self-care skills, and health behaviors. To the extent that disease knowledge, self-care skills and health behaviors are important determinants for health, examining their associations with health literacy may help to devise interventions to improve the health of disadvantaged subgroups of the population.

## Conclusions

From our study it is evident that low health literacy is widespread even in countries such as Taiwan that have a high rate of literacy. This finding lends support to the claim that low health literacy may be prevalent around the world. However, the prevalence itself is not necessarily indicative of the need for interventions. As our results suggest, the health effects of low health literacy may vary as a result of the socio-demographic composition of the population and the structure of the health care delivery and financing systems. To the extent this is true, interventions that focus solely on enhancing health literacy may have a limited impact on health and health care utilization in certain countries. Further research that examines the impact of low health literacy on health outcomes across countries would help to verify our contention and inform health policy in other countries.

## Competing interests

The authors declare that they have no competing interests.

## Authors' contributions

SYDL conceived of the study, participated in the design of the study, interpretation of the data, and led the manuscript preparation. TIT conceived of the study, participated in the design of the study, carried out the survey and analysis, interpretation of the data, and involve in drafting the manuscript. YWT participated in the design of the study, carried out the survey, and participated in manuscript preparation. KNK participated in the survey design and coordination. All authors read and approved the final manuscript.

## Pre-publication history

The pre-publication history for this paper can be accessed here:

http://www.biomedcentral.com/1471-2458/10/614/prepub
